# Clustering-derived clinical subtypes and outcomes of chronic kidney disease in patients with diabetes: a multicenter cohort study

**DOI:** 10.3389/fendo.2026.1866115

**Published:** 2026-07-23

**Authors:** Jeong-Yeun Lee, Hyunjee Kim, Dong Keon Yon, Jung Pyo Lee, Sang Youl Rhee, Soie Kwon, Seokwoo Park, Yaerim Kim, Hyeon Seok Hwang

**Affiliations:** 1 Division of Nephrology, Department of Internal Medicine, College of Medicine, Kyung Hee University, Kyung Hee University Medical Center, Seoul, Republic of Korea; 2 Center for Digital Health, Medical Science Research Institute, Kyung Hee University College of Medicine, Seoul, Republic of Korea; 3 Department of Precision Medicine, Kyung Hee University School of Medicine, Seoul, Republic of Korea; 4 Department of Pediatrics, Kyung Hee University Medical Center, Kyung Hee University College of Medicine, Seoul, Republic of Korea; 5 Department of Internal Medicine–Nephrology, Seoul National University Boramae Medical Center, Seoul, Republic of Korea; 6 Department of Endocrinology and Metabolism, Kyung Hee University College of Medicine, Seoul, Republic of Korea; 7 Department of Internal Medicine–Nephrology, Chung-Ang University Hospital, Seoul, Republic of Korea; 8 Department of Internal Medicine–Nephrology, Seoul National University Bundang Hospital, Seongnam, Gyeonggi-do, Republic of Korea; 9 Department of Internal Medicine–Nephrology, Keimyung University Dongsan Medical Center, Daegu, Republic of Korea

**Keywords:** chronic kidney disease, clustering analysis, diabetes, end-stage kidney disease, subtyping

## Abstract

**Background:**

Chronic kidney disease (CKD) with diabetes is a heterogeneous condition with multiple underlying causes. However, no classification system appropriately captures the diversity of patients with CKD and diabetes or provides prognostic insights.

**Methods:**

We included 11,925 patients with CKD and diabetes from the Multicenter Cohort of Diabetic Kidney Disease Study (MEET-DKD) between 2000–2023. K-means clustering was implemented using thirty-two variables to identify the subtypes of CKD with diabetes. The associations between subtypes and cardiovascular events, progression to end-stage kidney disease (ESKD), and mortality were evaluated.

**Results:**

Three distinct diabetic CKD subgroups were identified; cluster 1 (n=4,494 [37.7%]) included predominantly older patients with multiple comorbidities and the highest medication use (comorbidity-dominant subtype); cluster 2 (n = 2,240 [18.8%]) included patients with lower renal function, and a moderate burden of comorbidities and medications (advanced CKD subtype); and cluster 3 (n = 5,191 [43.5%]) comprised patients with a higher prevalence of obesity, fewer comorbidities, and comparatively favorable laboratory profiles (obesity-dominant subtype). Compared with the obesity-dominant subtype, the comorbidity-dominant subtype exhibited the highest mortality risk (hazard ratio, 1.48; 95% confidence interval, 1.37–1.59). For progression to ESKD, using the comorbidity-dominant subtype as the reference, the advanced CKD subtype showed the highest risk (hazard ratio, 1.46; 95% confidence interval, 1.31–1.64). Similarly, compared with the comorbidity-dominant subtype, the obesity-dominant subtype showed the greatest risk of cardiovascular events (hazard ratio, 1.47; 95% confidence interval, 1.32–1.64).

**Conclusion:**

Our study identified three distinct subgroups of diabetic patients with CKD with differential risks for major outcomes. This highlights the heterogeneity of CKD in patients with diabetes, and suggest that identified subtypes may provide additional prognostic information for risk stratification. Further external validation is required before clinical implementation.

## 1 Introduction

Chronic kidney disease (CKD) is one of the most severe long-term complications of diabetes and is becoming increasingly important in patients with diabetes (1, 2). As the global prevalence of diabetes increases, it is being recognized as a leading cause of CKD. Approximately 20%–40% of individuals with diabetes are estimated to develop CKD, and those with both diabetes and CKD account for nearly 50% of patients receiving renal replacement therapy (3, 4). In addition, both diabetes and CKD are significant risk factors for cardiovascular (CV) disease and all-cause mortality, and their coexistence has a synergistic effect on these adverse clinical outcomes (5, 6). Therefore, the high prevalence and comorbidity burden of diabetes-related CKD poses major challenges to healthcare systems (7).

Hyperglycemia plays a central role, triggering oxidative stress and inflammatory responses, and increasing the risk of kidney injury (8, 9). However, this alone does not fully explain the clinical phenotypes observed in this population. A range of interrelated factors, including increased blood pressure, dyslipidemia, CV morbidity, and therapeutic agents, also contribute significantly to kidney damage (10, 11). This pathophysiological heterogeneity is directly reflected in the clinical setting, leading to wide variations in manifestations at the time of CKD presentation. Therefore, patients present with different baseline characteristics, comorbidity burdens, and risks of adverse outcomes (12, 13). However, the current CKD classification system grades disease severity based on glomerular filtration rate and albuminuria (14). This framework, identical to that used for non-diabetic CKD, fails to capture the unique characteristics and complexity of CKD in patients with diabetes. Consequently, patients within the same KDIGO category may exhibit substantial heterogeneity in metabolic profiles, comorbidity burden, disease progression, and clinical outcomes. Recent advances in precision nephrology have highlighted the limitations of conventional classification systems and emphasized the need to identify clinically meaningful patient subgroups not fully captured by traditional risk categories. Artificial intelligence and machine learning approaches have emerged as promising strategies for capturing the multidimensional heterogeneity of CKD and facilitating more individualized risk assessment and management (15, 16).

Therefore, this study classified patients into clusters incorporating major clinical characteristics from a multicenter cohort of patients with CKD and diabetes. We aimed to identify distinct subgroups within this population, based on clinical parameters and phenotypes. Subsequently, we evaluated whether these data-driven clusters exhibited differential risks for adverse renal and CV events and all-cause mortality to provide a basis for more individualized management strategies.

## 2 Methods

### 2.1 Study population

This multicenter cohort study used the Multicenter Cohort of Diabetic Kidney Disease (MEET DKD) database, which includes patients with CKD enrolled from six medical centers across South Korea. Eligible participants were adults aged ≥18 years who met at least one of the following criteria: (i) proteinuria ≥300 mg/g or persistent microalbuminuria ≥30 mg/g for more than 3 months; and (ii) estimated glomerular filtration rate (eGFR) <60 mL/min/1.73 m² for more than 3 months, and an eGFR of at least 30 mL/min/1.73 m² at the time of cohort entry. The 2009 Chronic Kidney Disease Epidemiology Collaboration equation (CKD-EPI) was used to calculate eGFR (17). Patients were retrospectively identified between January 2000 and December 2023. The time of cohort entry was defined as the time at which the participants first met the CKD eligibility criteria. A total of 38,710 individuals were initially screened, and 12,208 patients with diabetes were identified. After excluding 135 individuals with missing dates, 144 with missing body mass index (BMI) value, and 4 aged <20 years, 11,925 CKD patients with diabetes were included in the final analysis (**Supplementary Figure 1**). The study protocol was approved by the Institutional Review Board of each participating center (KHUH 2023-06-010-001).

### 2.2 Data collection

The MEET DKD database collected data at the time of cohort enrollment on parameters including demographic information, laboratory findings, and medications in use. A total of 32 variables were selected based on their capacity to represent both prognostic and clinical aspects of CKD (18–21). These variables included demographic characteristics (age, sex, and BMI), comorbid conditions (hypertension, dyslipidemia, history of CV disease, and history of cancer), laboratory findings (eGFR, urine protein-to-creatinine ratio [UPCR], hemoglobin, white blood cell [WBC] count, platelet count, serum albumin, hemoglobin A1c [HbA1c], serum sodium, potassium, calcium, phosphate, uric acid, glucose, aspartate aminotransferase [AST], alanine aminotransferase [ALT], and low-density lipoprotein cholesterol [LDL]), and medications in current use extracted from electronic medical records (metformin [MFM], sulfonylureas [SU], dipeptidyl peptidase-4 inhibitors [DPP-4i], insulin, angiotensin receptor blockers [ARBs] or angiotensin-converting enzyme inhibitors [ACEi], diuretics, β-blockers, statins, and antiplatelet agents).

### 2.3 Study end points

Our primary objective was to identify well-defined subgroups of the study population with differential characteristics. Secondary outcomes were ascertained from the Multicenter Cohort of Diabetic Kidney Disease Study (MEET-DKD) database and included cardiovascular (CV) disease, progression to end-stage kidney disease (ESKD), and all-cause mortality. CV disease was defined as a composite of ischemic heart disease, myocardial infarction, and stroke, identified using ICD-10 codes and corresponding procedure codes as detailed in **Supplementary Table 1**. Progression to ESKD was defined as the initiation of renal replacement therapy, including hemodialysis, peritoneal dialysis, or kidney transplantation, and was identified using diagnostic codes, procedure codes, and insurance codes for dialysis (22, 23). All-cause mortality was identified from the database records and ascertained through linkage with mortality records from Statistics Korea (24, 25). Participants were followed until death, loss to follow-up, or September 18, 2023.

### 2.4 Clustering analysis and evaluation

K-means clustering analysis was performed to categorize the patients with chronic kidney disease and diabetes into clusters based on their baseline clinical data (26). This unsupervised machine learning algorithm partitions the data into K distinct clusters by grouping observations with similar characteristics (27). K-means clustering was used as the primary clustering method because this study aimed to identify clinically interpretable phenotypic profiles based on similarities across standardized baseline characteristics. The centroid-based structure of K-means enabled each cluster to be summarized as an average clinical profile, thereby facilitating clinical characterization and labeling of patient subtypes. Because of the large sample size of the present cohort, K-means also provided a computationally efficient and transparent approach for exploratory clinical phenotyping.

Before clustering, all baseline variables were converted into numeric input features. Continuous variables were retained as continuous measures, whereas binary variables, including sex, comorbidities, and medication use, were encoded as indicator variables. All encoded baseline variables were included in the clustering process after standardization using Z-score transformation to ensure comparability across different measurement scales. Therefore, each input variable contributed equally to the Euclidean distance calculation after standardization, and no additional variable-specific weighting was applied. K-means clustering was performed using multiple random initializations with a fixed random seed to ensure reproducibility.

To assess potential redundancy among clustering variables, pairwise Spearman correlation coefficients were calculated using the complete-case analytic dataset. Highly correlated variable pairs were defined as those with an absolute rho of 0.80 or greater. No variable pairs showed an absolute Spearman rho of 0.80 or greater.

The optimal number of clusters was determined by combining the elbow method, the adjusted Rand index (ARI) derived from bootstrap resampling, and the Davies-Bouldin index (28). Specifically, the optimal cluster count was identified at the point where the within-cluster variance curve from the elbow method began to stabilize and the mean ARI across bootstrap iterations reached its maximum. Dimensionality reduction and visualization of the clustering results were performed using principal component analysis (PCA) to illustrate the distinct spatial distribution of the identified clusters (29). Internal validation of clustering stability was assessed using a subsampling consensus heatmap that evaluated the consistency of cluster membership across multiple bootstrap resamples. Furthermore, intermodel validation was performed by comparing the K-means clustering results with those obtained using hierarchical clustering. To evaluate whether medication variables influenced cluster assignment, we performed a sensitivity analysis with medication variables excluded from the K-means clustering input features while preserving the original grouped-variable preprocessing strategy. The medication-excluded clustering solution was compared with the primary K-means clustering solution using cross-tabulation. Comprehensive methodological details for dimensionality reduction, subsampling consensus heatmap analysis, inter-model validation using hierarchical clustering, and medication-excluded clustering sensitivity analysis are provided in the **Supplementary Methods**.

### 2.5 Statistical analyses

Continuous variables are expressed as mean ± standard deviation, and categorical variables are expressed as numbers and percentages. Baseline characteristics and differences between groups were analyzed using the chi-square test for categorical variables and analysis of variance for continuous variables. For the medication-excluded sensitivity analysis, categorical variables were compared using the chi-square test, and continuous variables were compared using the Kruskal-Wallis test. Incidence rates were presented as the number of events per 1,000 person-years. The associations between each defined cluster and clinical outcomes were evaluated using outcome-specific survival models. Fine–Gray sub-distribution hazard models were used to analyze CV events and progression to ESKD, with death before the occurrence of each outcome treated as a competing event. Sub-distribution hazard ratios with 95% confidence intervals were reported. Censoring was assumed to be non-informative. All-cause mortality was analyzed using Cox proportional hazards regression models because death itself was the outcome of interest. Models were fitted sequentially from an unadjusted model to multivariable models adjusted for demographic factors, comorbidities, kidney function, laboratory parameters, serum albumin, UPCR/proteinuria, and enrollment period.

Cumulative event rates for each adverse clinical outcome are presented using Kaplan-Meier plots with log-rank tests for descriptive visualization. One-, three-, and five-year cumulative incidence estimates and absolute risk differences were additionally calculated for each clinical outcome by subtype, using the comorbidity-dominant subtype as the reference group; for CV events and progression to ESKD, death was considered as a competing event in cumulative incidence estimation (**Supplementary Methods**). The proportional hazards assumption was assessed using Schoenfeld residual tests. Because the assumption was not fully satisfied for several cluster-outcome associations, exploratory time-stratified Cox regression analyses were performed before and after 7 years of follow-up. Net reclassification improvement (NRI), integrated discrimination improvement (IDI), and 5-year time-dependent AUC were calculated to assess the incremental predictive value of adding diabetic CKD subtype to Model 3 (**Supplementary Methods**).

Statistical significance was defined as a two-sided P value <0.05. All statistical analyses were performed using SAS (version 9.4; SAS Institute, Cary, NC, USA), R (version 3.4.1; The R Foundation for Statistical Computing, Vienna, Austria; http://www.R-project.org), or Python (version 3.12.7; Python Software Foundation, https://www.python.org). The overall analytical workflow is summarized in **Supplementary Figure 2**.

## 3 Results

### 3.1 Identification of CKD subtypes in diabetes

**Figure 1:**
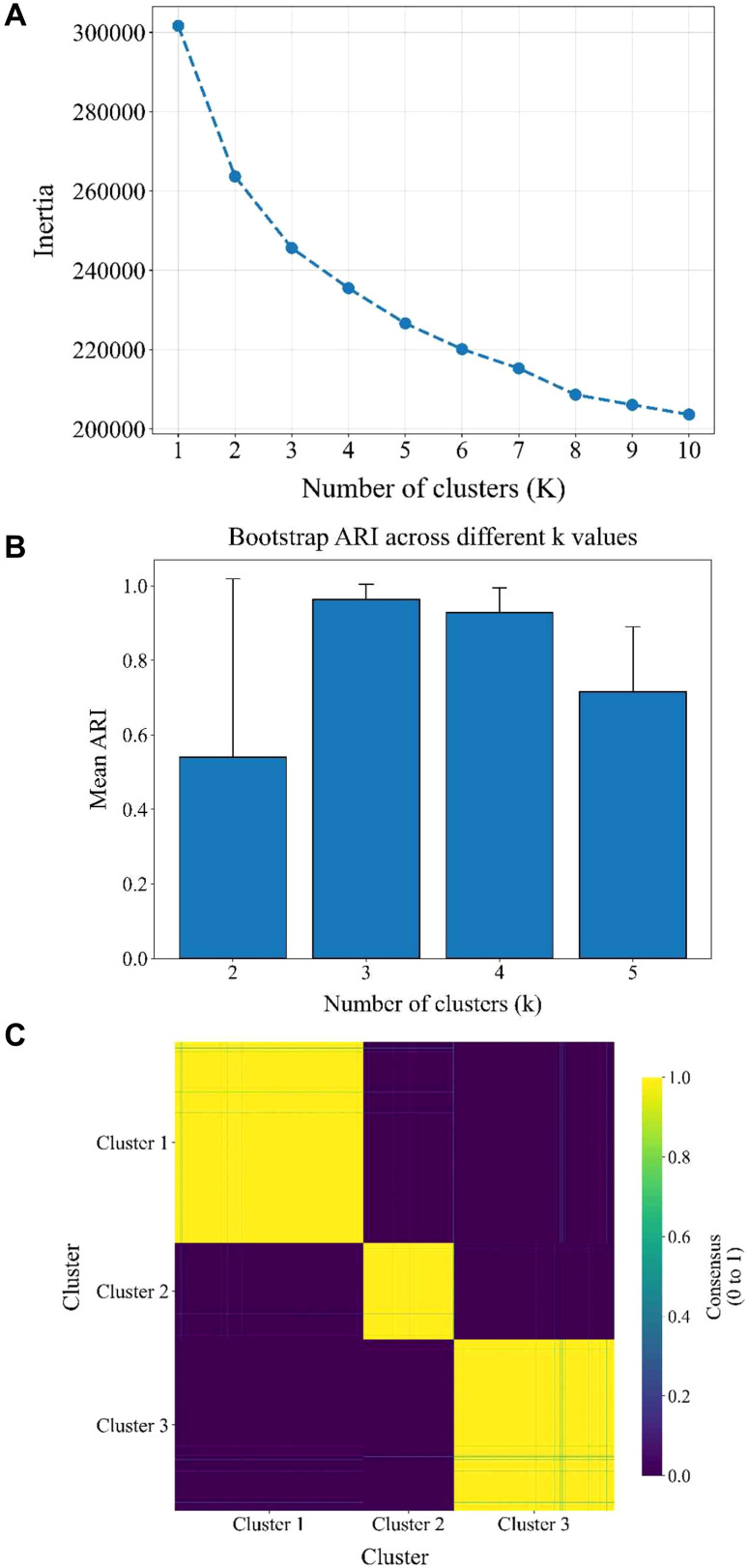
Determining the optimal number of clusters in patients with diabetes: **(A)** elbow method analysis, **(B)** bootstrap-based ARI for k = 2, 3, 4, and 5, **(C)** subsampling consensus heatmap. ARI, adjusted Rand index.

K-means clustering based on 32 selected variables categorized patients with CKD and diabetes into three distinct clusters. Of the 11,925 participants, 4,494 (37.7%), 2,240 (18.8%), and 5,191 (43.5%) were assigned to clusters 1, 2, and 3, respectively. The elbow method was used to determine the optimal number of clusters. Inertia decreased sharply from 301,650 with one cluster (k = 1) to 263,665 with two clusters (k = 2), and further to 245,621 with three clusters (k = 3), after which the decline became more gradual (**Figure 1A**). In the bootstrap analysis, the ARI was 0.540 ± 0.478 at two clusters, reached its highest value at three clusters (0.963 ± 0.041), and then declined at higher solutions, with 0.928 ± 0.067 at four clusters and 0.715 ± 0.175 at five clusters (**Figure 1B**). The consensus heatmap analysis demonstrated strong within-cluster stability and low between-cluster overlap (**Figure 1C**). Diagonal blocks corresponding to clusters 1, 2, and 3 were uniformly high (yellow), indicating that patients assigned to the same cluster were consistently grouped in the same cluster across repeated subsampling. The mean consensus scores were 0.986 for cluster 1, 0.991 for cluster 2, and 0.992 for cluster 3. In addition, cluster validity was evaluated using the Davies–Bouldin index, which also supported a three-cluster solution as the optimal clustering structure (**Supplementary Figure 3**).

### 3.2 Validation of clustering results

A PCA plot was used to visualize and assess the distinctness of the identified clusters. The PCA plot demonstrated distinct clustering patterns with limited overlap among the identified subtypes, supporting the validity of K-means clustering and indicating that the identified subtypes captured the major axes of variation in the clinical data (**Supplementary Figure 4**). Intermodel validation was conducted by comparison with hierarchical clustering to assess the reliability of the clustering solution (**Supplementary Table 2**). In hierarchical clustering, the majority of patients in K-means cluster 1 were also assigned to cluster B (82.1%), whereas most of those in cluster 2 were classified as cluster A (72.6%), and cluster 3 showed close alignment with cluster C (69.1%). The agreement between K-means and other clustering methods is visualized in **Supplementary Figure 5**.

We repeated K-means clustering after excluding medication variables from the input features. The medication-excluded clustering solution showed generally consistent patterns with the primary K-means clustering solution (**Supplementary Table 3**, **S4**).

### 3.3 Baseline characteristics of subtypes

The baseline characteristics of clusters 1, 2, and 3 are summarized in **Table 1**. Cluster 1 included the highest proportion of patients aged ≥70 years among the three clusters. Cluster 3 had more men and individuals with BMI ≥25 kg/m^2^. Cluster 1 exhibited a greater prevalence of hypertension, dyslipidemia, and a history of CV disease; cluster 2 showed a moderate burden of comorbidities; and cluster 3 was associated with fewer comorbidities. Cluster 2 included more patients with reduced eGFR and proteinuria and also revealed lower mean levels of hemoglobin and albumin, along with higher mean leukocyte counts. This cluster was also associated with hypocalcemia, hyperphosphatemia, hyponatremia, and hyperuricemia compared with the others. Oral antidiabetic agents, antihypertensive agents, statins, and antiplatelet agents were most frequently prescribed in cluster 1. Cluster 2 exhibited intermediate levels of medication use, whereas cluster 3 was characterized by a generally lower use across all medication classes. Based on these distinguishing clinical features, the three clusters were labeled as the comorbidity-dominant, advanced CKD, and obesity-dominant subtypes, respectively. The variables contributing most prominently to the distinction among the three subtypes are summarized in **Supplementary Figure 6**.

**Table 1:** Baseline characteristics of the study population.

Characteristics	CKD in diabetes subtypes			
	Comorbidity-dominant subtype (n = 4,494)	Advanced CKD subtype (n = 2,240)	Obesity-dominant subtype (n = 5,191)	*P* value
Demographics, n (%)				
Age				
19-29, n (%)	5 (0.1)	23 (1.0)	50 (1.0)	<0.001
30-39, n (%)	60 (1.3)	79 (3.5)	102 (2.0)	
40-49, n (%)	231 (5.1)	220 (9.8)	310 (6.0)	
50-59, n (%)	661 (14.7)	437 (19.5)	836 (16.1)	
60-69, n (%)	1,445 (32.2)	574 (25.6)	1,728 (33.3)	
≥70, n (%)	2,092 (46.6)	907 (40.5)	2,165 (41.7)	
Male	2,489 (55.4)	1,232 (55.0)	3,106 (59.8)	<0.001
BMI (kg/m²)				
<18.5, n (%)	69 (1.5)	122 (5.5)	127 (2.5)	<0.001
≥18.5 and < 23, n (%)	1,401 (31.2)	843 (37.6)	1,521 (29.3)	
≥23 and < 25, n (%)	1,836 (40.9)	692 (30.9)	1,761 (33.9)	
≥25, n (%)	1,188 (26.4)	583 (26.0)	1,782 (34.3)	
Comorbidity, n (%)				
Hypertension, n (%)	4,315 (96.0)	1,272 (56.8)	1,752 (33.8)	<0.001
Dyslipidemia, n (%)	3,673 (81.7)	745 (33.3)	1,241 (23.9)	<0.001
CVD, n (%)	1,098 (24.4)	264 (11.8)	916 (17.7)	<0.001
Cancer, n (%)	218 (4.9)	105 (4.7)	506 (9.8)	<0.001
Laboratory data, n (%)				
eGFR (mL/min/1.73 m²)				
≥60, n (%)	897 (20.0)	247 (11.0)	839 (16.2)	<0.001
45-60, n (%)	2,098 (46.7)	537 (24.0)	2,605 (50.2)	<0.001
<45, n (%)	1,499 (33.4)	1,456 (65.0)	1,747 (33.7)	<0.001
UPCR ≥300 mg/g	2,461 (54.8)	2,035 (90.9)	3,329 (64.1)	<0.001
Laboratory data, mean (SD)				
HbA1c (%)	7.2 ± 1.3	7.9 ± 2.2	7.4 ± 1.6	<0.001
Hb (g/dL)	12.3 ± 2.0	10.0 ± 1.8	12.4 ± 1.9	<0.001
Leukocyte (10^3^/uL)	7.5 ± 2.6	9.6 ± 7.2	7.4 ± 2.6	<0.001
Platelet (10^3^/uL)	241.5 ± 79.7	240.0 ± 114.4	239.4 ± 86.6	0.015
Albumin (g/dL)	4.1 ± 0.4	3.2 ± 0.5	4.1 ± 0.4	<0.001
Sodium (mEq/L)	139.6 ± 3.2	137.1 ± 5.5	139.8 ± 3.4	<0.001
Potassium (mEq/L)	4.5 ± 0.6	4.6 ± 0.8	4.6 ± 0.6	0.544
Chloride (mEq/L)	104.2 ± 4.1	104.6 ± 6.6	104.6 ± 4.3	<0.001
Calcium (mg/dL)	9.0 ± 0.5	8.3 ± 0.7	9.2 ± 0.6	<0.001
Phosphorus (mg/dL)	3.6 ± 0.7	4.0 ± 1.4	3.6 ± 0.7	<0.001
Uric acid (mg/dL)	6.4 ± 2.0	7.2 ± 2.6	6.3 ± 1.9	<0.001
Glucose (mg/dL)	147.9 ± 66.3	192.8 ± 125.4	150.7 ± 73.9	<0.001
AST (U/L)	24.8 ± 13.8	29.6 ± 27.3	24.1 ± 13.0	<0.001
ALT (U/L)	21.6 ± 13.1	21.6 ± 15.8	21.8 ± 13.5	0.249
LDL (mg/dL)	88.0 ± 32.9	105.1 ± 51.1	96.2 ± 34.4	<0.001
Medication, n (%)				
MFM, n (%)	2,559 (56.9)	101 (4.5)	261 (5.0)	<0.001
SU, n (%)	1,999 (44.5)	162 (7.2)	297 (5.7)	<0.001
DPP4i, n (%)	1,351 (30.1)	114 (5.1)	101 (2.0)	<0.001
Insulin, n (%)	459 (10.2)	292 (13.0)	102 (2.0)	<0.001
ARB or ACEi, n (%)	3,460 (77.0)	358 (16.0)	200 (3.9)	<0.001
Diuretics, n (%)	1,741 (38.7)	562 (25.1)	220 (4.2)	<0.001
Beta blocker, n (%)	1,380 (30.7)	224 (10.0)	41 (0.8)	<0.001
Statin, n (%)	3,292 (73.3)	253 (11.3)	145 (2.8)	<0.001
Anti-platelet, n (%)	3,331 (74.1)	362 (16.2)	310 (6.0)	<0.001

ACEi, angiotensin converting enzyme inhibitor; ALT, alanine aminotransferase; ARB, angiotensin II receptor blocker; AST, aspartate aminotransferase; BMI, body mass index; Ca, calcium; Cl, chloride; CVD, cardiovascular disease; DPP4i, dipeptidyl peptidase 4 inhibitor; eGFR, estimated glomerular filtration rate; Hb, hemoglobin; HbA1c, hemoglobin A1c; LDL, low density lipoprotein cholesterol; MFM, metformin; SU, sulfonylurea; UPCR, urine protein to creatinine ratio.

* A P-value <0.001 indicates statistically significant difference among the clusters.

### 3.4 Clinical outcomes based on identified subtype

**Figure 2:**
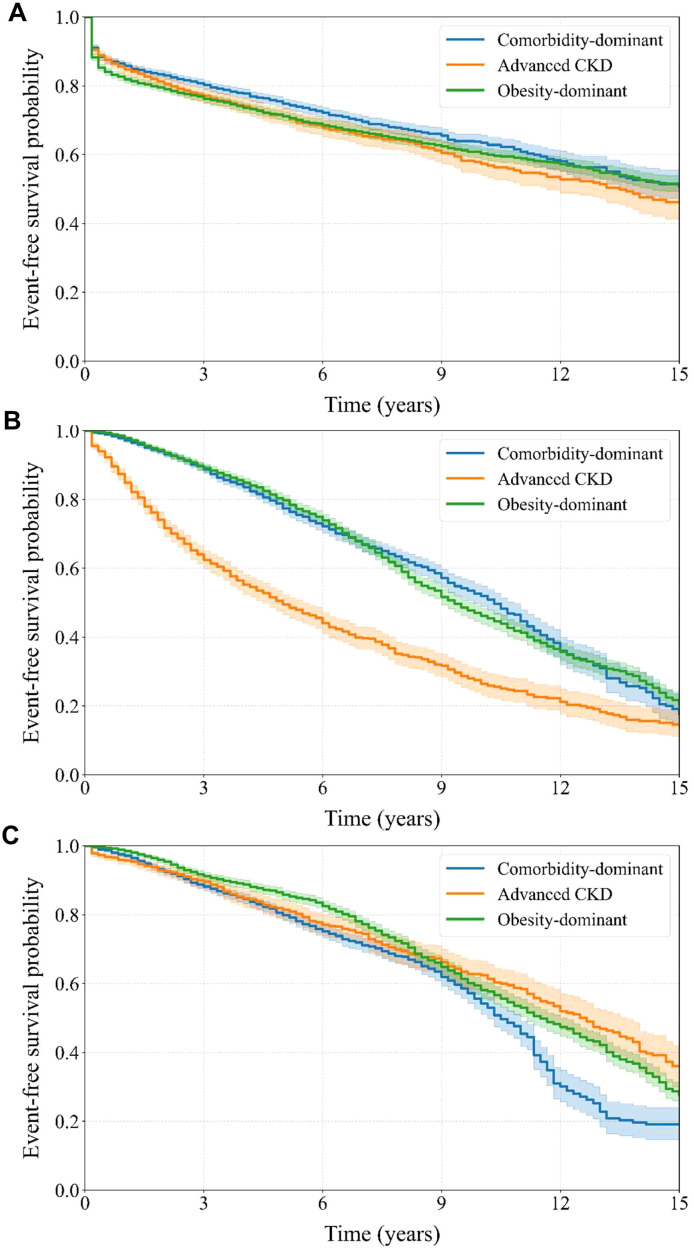
Kaplan–Meier curve of **(A)** CV disease, **(B)** progression to ESKD, and **(C)** all-cause mortality. CVD, cardiovascular disease; ESKD, end-stage kidney disease.

We analyzed adverse clinical outcomes according to the categorized subtypes of diabetic CKD. During a median follow-up period of 5.42 years, a total of 3,485 cases of CV disease, 4,390 cases of progression to ESKD, and 6,702 deaths from all causes were documented. Age-standardized all-cause mortality rates were calculated to provide context for the mortality burden across subtypes (**Supplementary Table 5**). The Kaplan–Meier curves demonstrated a significantly different incidence of clinical outcomes among the three identified clusters (**Figure 2**). The advanced CKD subtype exhibited poorer event-free survival over time for CV events than the other clusters. This subtype was also associated with a significantly higher cumulative event rate of progression to ESKD. Meanwhile, the cumulative event rate for all-cause mortality was highest in comorbidity-dominant subtype. Numerical estimates of one-, three-, and five-year cumulative incidence further quantified the absolute risk burden across the diabetic CKD subtypes (**Supplementary Table 6**).

**Table 2** shows the number of events, incidence rates in person-years, and hazard ratios (HRs) for CV events, progression to ESKD, and all-cause mortality. In univariate analysis, obesity-dominant subtype was associated with the greatest risk of CV disease, and advanced CKD subtype showed a higher risk of progression to ESKD compared to comorbidity-dominant subtype. These associations were maintained after adjusting for multiple covariates. Compared with comorbidity-dominant subtype, the adjusted HRs (95% confidence interval [CI]) for CV disease were 1.47 (1.32–1.64) for the obesity-dominant subtype and 1.34 (1.18–1.52) for the advanced CKD subtype, respectively. Advanced CKD and obesity-dominant subtypes demonstrated substantially elevated risk of progression to ESKD compared to comorbidity-dominant subtype, and advanced CKD subtype showed the greatest risk among three clusters (adjusted HR 1.46, 95% CI 1.31–1.64). Meanwhile, comorbidity-dominant and advanced CKD subtypes were associated with greater risk of all-cause mortality, while obesity-dominant subtype exhibited a significantly lower risk as follows: adjusted HR with 95% CI 1.48 (1.37–1.59) for comorbidity-dominant, and 1.46 (1.35–1.57) for advanced CKD subtype compared to obesity-dominant subtype.

**Table 2:** Incidence and hazard ratios of CV disease, progression to ESKD, and all-cause mortality based on identified clusters.

Outcomes	Number of events	Person year	Incidence rate	HR or sHR (95% CI)		
				Model 1	Model 2	Model 3
CV disease						
Comorbidity-dominant	1,157	32,604	35.49	Ref	Ref	Ref
Advanced CKD	607	13,974	43.44	**1.12** **(1.02 – 1.24)**	**1.42** **(1.26 – 1.60)**	**1.23** **(1.06 - 1.42)**
Obesity-dominant	1,721	38,256	44.99	**1.32** **(1.22 – 1.42)**	**1.49** **(1.34 – 1.66)**	**1.42** **(1.27 - 1.58)**
Progression to ESKD						
Comorbidity-dominant	1,194	36,765	32.48	Ref	Ref	Ref
Advanced CKD	900	12,410	72.52	**1.95** **(1.77 – 2.13)**	**1.65** **(1.49 – 1.83)**	**1.31** **(1.15 - 1.49)**
Obesity-dominant	2,296	46,746	49.12	**1.52** **(1.42 – 1.62)**	**1.28** **(1.17 – 1.40)**	**1.23** **(1.12 - 1.36)**
All-cause mortality						
Comorbidity-dominant	2,045	23,937	85.43	Ref	Ref	Ref
Advanced CKD	1,254	11,691	107.26	1.04 (0.97 – 1.12)	**1.19** **(1.10 – 1.29)**	**0.76** **(0.69 - 0.84)**
Obesity-dominant	3,403	41,410	82.18	**0.73** **(0.69 – 0.77)**	**0.68** **(0.63 – 0.73)**	**0.67** **(0.62 - 0.72)**

CI, confidence interval; CV, cardiovascular; ESKD, end-stage kidney disease; HR, hazard ratio; sHR, subdistribution hazard ratio.

Model 1: unadjusted.

Model 2: adjusted for age, sex, BMI, hypertension, dyslipidemia, history of CV disease, history of cancer.

Model 3: adjusted for age, sex, BMI, hypertension, dyslipidemia, history of CV disease, history of cancer, eGFR, Hb, HbA1c, LDL, Albumin, UPCR, and enrollment period.

*The values in bold font represent a significant variance (p < 0.05).

*Incidence rate per 1,000 person-year

*Death was treated as competing event for CV disease and progression to ESKD in Fine-Gray models.

*sHRs from Fine–Gray subdistribution hazard models are reported for CV disease and progression to ESKD, with death treated as a competing event. HRs from Cox proportional hazards models are reported for all-cause mortality.

Schoenfeld residual tests indicated that the proportional hazards assumption was not fully satisfied for several cluster-outcome associations (**Supplementary Table 7**). Therefore, exploratory time-stratified Cox regression analyses were performed before and after 7 years of follow-up. These analyses suggested that the elevated risks of cardiovascular disease and ESKD progression in specific subtypes were mainly observed during the first 7 years, whereas the comorbidity-dominant subtype showed consistently higher all-cause mortality across both time periods (**Supplementary Table 8**).

### 3.5 Predictive performance of clustering-based subtypes

**Figure 3:**
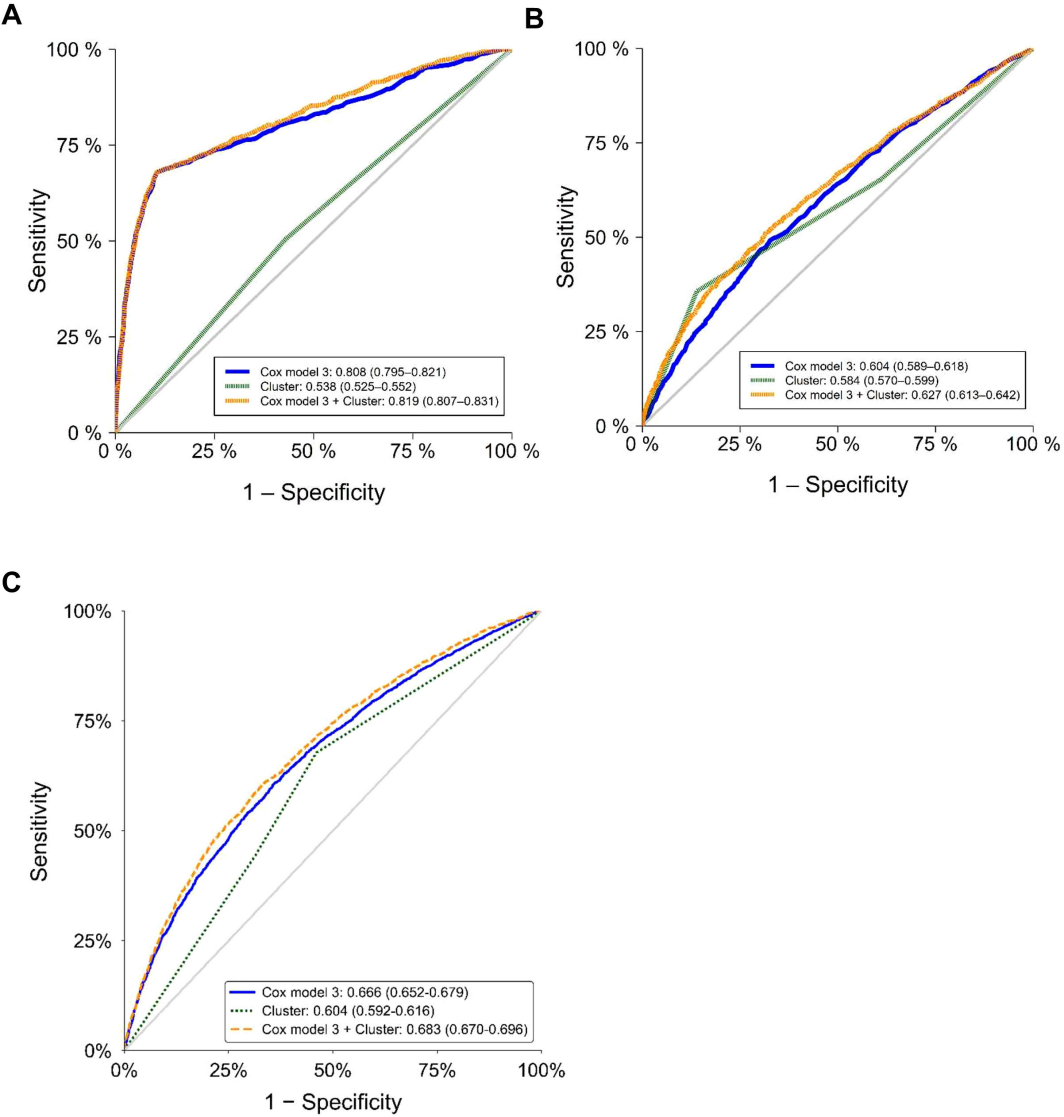
Time-dependent ROC curves at 5-year follow-up for prediction of **(A)** CV disease occurrence, **(B)** progression to ESKD, and **(C)** all-cause mortality. ROC, Receiver Operating Characteristic; CVD, cardiovascular disease; ESKD, end-stage kidney disease. * Both model comparisons (Cox model 3 vs. Cox model 3 + Cluster; Cluster-only vs. Cox model 3 + Cluster) were statistically significant (p < 0.01) across all three outcomes. * Time-dependent ROC curves for CV disease and ESKD were derived from Fine–Gray competing risk models, whereas those for all-cause mortality were based on Cox proportional hazards models.

To evaluate the predictive value of our clustering results, we performed time-dependent ROC curve analysis. In the 5-year prediction of CV disease, the AUC (95% CI) was 0.808 (0.795–0.821) for Cox model 3 alone and increased to 0.819 (0.807–0.831) when cluster information was incorporated (**Figure 3A**). In the analysis of ESKD progression, the time-dependent AUC was 0.604 (0.589–0.618) for Cox model 3 and improved to 0.627 (0.613–0.642) with the addition of cluster information (**Figure 3B**). A similar pattern was observed for all-cause mortality as the time-dependent AUC increased from 0.676 (0.663–0.690) for Cox model 3 to 0.695 (0.683–0.709) with the inclusion of cluster information (**Figure 3C**). All improvements in predictive performance from the Cox model alone to the Cox model incorporating cluster information were statistically significant (all *P* < 0.01). To further evaluate the incremental predictive value of the identified subtypes, we additionally calculated the NRI and IDI for 5-year risk prediction (**Supplementary Table 9**). The addition of cluster information significantly improved IDI for all outcomes including CV disease (IDI 0.002, 95% CI 0.001–0.003; P = 0.001), progression to ESKD (IDI 0.006, 95% CI 0.004–0.007; P = 0.001), and all-cause mortality (IDI 0.007, 95% CI 0.006–0.009; P = 0.001). Significant improvements in NRI were also observed for progression to ESKD (NRI 0.111, 95% CI 0.057–0.164; P = 0.001) and all-cause mortality (NRI 0.293, 95% CI 0.245–0.339; P = 0.001), whereas the NRI for CV disease was not statistically significant (NRI 0.021, 95% CI −0.030 to 0.072; P = 0.342).

## 4 Discussion

In this study, we introduced the K-means clustering algorithm to baseline variables and identified three distinct clusters. These clusters represented clinically meaningful subgroups that captured multidimensional patterns in the baseline characteristics. The cluster structure demonstrated strong internal consistency and reliability across multiple validation approaches. Furthermore, each cluster exhibited notable differences in age, comorbidities, obesity status, and kidney function and showed significant differences in the risks of CV events, progression to ESKD, and all-cause mortality. These findings suggest that characterization of this heterogeneity is a key step in individualizing follow-up strategies for patients with CKD and diabetes.

We found that patients in comorbidity-dominant subtype had older age, a high burden of comorbidities, and frequent use of multiple medications. Chronic hypertension, dyslipidemia, and widespread atherosclerosis may contribute to cumulative systemic insults and target organ damage, suggesting that the development of CKD in this cluster may result from the systemic impact of multiple comorbid conditions (30–32). We also found that patients in comorbidity-dominant subtype had a higher risk of all-cause mortality than those in obesity-dominant subtype, which may be attributed to their older age and the burden of multiple comorbidities. These findings suggest that the comorbidity-dominant subtype phenotype underscores the importance of the comprehensive management of comorbid conditions and optimization of medication use to improve clinical outcomes.

While patients in the comorbidity-dominant subtype showed a higher risk of all-cause mortality, the risks of CV disease and progression to ESKD followed different patterns. The cumulative incidence of CV disease and progression to ESKD was lower in comorbidity-dominant subtype, and the risks of both outcomes were the lowest in the competing risk analysis among the three clusters. These findings may reflect the higher mortality risk in this group, which might have reduced the opportunity of experiencing CV disease or ESKD progression (33, 34). Therefore, we suggest that mortality risk should be carefully considered when evaluating the risk of CV events or ESKD progression in patients with CKD categorized as comorbidity-dominant subtype.

In the present study, patients in advanced CKD subtype demonstrated lower eGFR levels along with associated manifestations such as anemia, hypoalbuminemia, hypocalcemia, and hyperphosphatemia, and also showed the highest prevalence of proteinuria (14). These findings suggest that the advanced CKD subtype represents a dominant feature of severe renal impairment. Interestingly, the baseline characteristics were generally intermediate between comorbidity-dominant and obesity dominant subtype in terms of comorbidity burden and medication use. Prognostic analyses also showed that advanced CKD subtype exhibited a risk of all-cause mortality similar to that of comorbidity-dominant subtype, and its risk of CV disease was closer to that observed in obesity-dominant subtype. These findings suggest that advanced CKD subtype represents a subtype sharing features of both phenotypes. Considering the progressive eGFR decline in CKD, it is plausible that some patients might have transitioned from comorbidity-dominant or obesity-dominant subtypes at earlier stages to advanced CKD subtype as their kidney function deteriorated.

Obesity-dominant subtype primarily included obese individuals with relatively preserved kidney function, fewer comorbidities, and less frequent medication use. This pattern suggests a metabolically driven, early-stage CKD phenotype preceding overt renal impairment. Interestingly, patients in obesity-dominant subtype showed a lower risk of all-cause mortality despite a substantially increased risk of CV disease and progression to ESKD. This apparent discrepancy may be partly consistent with the concept of the obesity paradox, where higher BMI is associated with a survival advantage in CKD (22, 35, 36). However, this finding should be interpreted cautiously, as patients in this cluster were generally younger and had fewer comorbidities. In addition, BMI-based obesity classification does not distinguish adiposity from lean mass or capture visceral adiposity, sarcopenia, nutritional status, or frailty. These unmeasured factors may have contributed to the observed results. Importantly, the relatively preserved survival in this cluster may represent a critical window of opportunity for intervention to prevent disease progression and improve long-term outcomes. Therefore, we suggest that patients with this CKD phenotype is the subgroup that may derive the greatest benefits from active management.

Prior clustering studies have examined subtypes of patients with CKD irrespective of diabetes status and have generally demonstrated a stepwise pattern, with worsening renal function, increasing comorbidity burden, and progressively higher risks of adverse clinical outcomes (37, 38). However, contrary to previous reports, the three clusters identified in our study did not simply reflect a stepwise deterioration in comorbidity burden, renal function, and risk of adverse outcomes. Instead, each cluster was defined according to distinct baseline characteristics, and the patterns of increased risk differed based on the type of adverse clinical events. This suggests that the three clusters maintained unique profiles, highlighting the heterogeneity of CKD in diabetes, and emphasizing the value of refined phenotyping for prognostic assessment. Importantly, incorporating these clustering-derived subtypes into risk prediction models improved the discriminative performance for clinical outcomes, underscoring their added prognostic value, in addition to conventional risk factors. These clustering-derived subtypes should be interpreted as prognostic clinical phenotypes rather than mechanistic disease subtypes. Nevertheless, they should not be directly implemented at the bedside. They may provide a framework for phenotype-based risk stratification using routinely available clinical variables. These subtypes may be useful to distinguish patients with different dominant risk profiles, such as CV events, ESKD progression, or mortality, and thereby support more individualized risk assessment and monitoring strategies. Future integration into electronic medical record-based prediction models may be considered after external validation, model simplification, and prospective evaluation.

Our study has several limitations. First, although K-means clustering provides a transparent and interpretable framework for clinical phenotyping, it has several methodological limitations. K-means clustering relies on Euclidean distance, which may not fully capture the complexity of mixed data types. Numeric encoding of categorical variables may introduce artificial distances between categories and may not fully reflect their clinical meaning. In addition, K-means clustering is sensitive to methodological choices, including feature selection, variable scaling, initialization, and the prespecified number of clusters. Second, K-means clustering is a data-driven approach that cluster membership depends on the characteristics of the input data. Several clinical variables, including smoking, blood pressure, diabetes duration, inflammatory markers, other classes of medications, were not consistently available throughout all participating centers and therefore could not be incorporated into the analysis. Third, although internal validation and inter-model validation supported the stability of the clustering results, external validation was not performed in the present study. Therefore, the reproducibility, transportability, and generalizability of the identified subtypes remain uncertain. Future studies should validate these phenotypes in independent external cohorts, including international populations with different ethnic backgrounds, healthcare systems, and treatment patterns. Fourth, as an observational cohort study, our analyses are inherently susceptible to potential residual confounding factors and treatment selection bias. Despite multivariate adjustment, unmeasured factors such as lifestyle, dietary habits, and medication adherence could have influenced both cluster membership and outcomes. In addition, because several clustering variables are also established prognostic factors, the potential influence of baseline disease severity on the observed associations cannot be fully excluded. Therefore, the clusters should be interpreted as data-driven clinical phenotypes with distinct prognoses, rather than as independent causal determinants of outcomes. Finally, treatment-era bias remains a potential concern because the long enrollment period spanned substantial changes in the management of CKD and diabetes. Changes in clinical practice, medication use, and supportive care over time may have influenced both cluster characteristics and clinical outcomes. Newer therapeutic agents such as sodium-glucose cotransporter-2 (SGLT2) inhibitors and glucagon-like peptide-1 receptor antagonists (GLP-1RAs) were used in only a small proportion of patients at baseline and therefore were not included in the clustering analysis.

In conclusion, our study identified three clinically distinct CKD subtypes in patients with diabetes, each associated with different risks of CV events, progression to ESKD, and all-cause mortality. This refined classification emphasizes the heterogeneity of CKD patients with diabetes and provides additional prognostic insights in addition to conventional risk factors. Clustering-derived subtypes may be useful to distinguish patients with different dominant risk profiles and support more individualized risk stratification and customized therapeutic strategies.

## Data Availability

The datasets presented in this article are not publicly available due to institutional and participant privacy restrictions. Requests to access the datasets should be directed to the corresponding author and are subject to approval by the participating institutions and relevant ethics committees.

## Supplementary material

Supplementary Table 1 ICD-10 codes and associated procedure codes used to identify CV diseases and ESKD

Supplementary Table 2 Matching matrix between different clustering model

Supplementary Table 3 Baseline characteristics of medication-excluded K-means clusters.

Supplementary Table 4 Agreement between original K-means clusters and medication-excluded feature-selected K-means clusters.

Supplementary Table 5 Age-standardized all-cause mortality rates according to diabetic CKD subtype.

Supplementary Table 6 One-, three-, and five-year cumulative incidence and absolute risk differences by diabetic CKD subtype, using the comorbidity-dominant subtype as the reference group.

Supplementary Table 7 Proportional hazards assumption assessment according to diabetic CKD subtype, using the comorbidity-dominant subtype as the reference group.

Supplementary Table 8 Time-stratified sensitivity analysis of clinical outcomes before and after 7 years of follow-up, using the comorbidity-dominant subtype as the reference group.

Supplementary Table 9 Incremental predictive performance after adding diabetic CKD subtype to Model 3.

Supplementary Figure 1 A flowchart of the study participant selection.

Supplementary Figure 2 Overall workflow of the study analysis

Supplementary Figure 3 Davies-Bouldin index according to the number of clusters.

Supplementary Figure 4 PCA plot of K-means clustering (k=3)

Supplementary Figure 5 Agreement of cluster assignments between K-means clustering and other clustering methods

Supplementary Figure 6 Standardized mean differences of baseline characteristics across the three diabetic CKD subtypes
