# Supplementary material for: Clustering-derived clinical subtypes and outcomes of chronic kidney disease in patients with diabetes: a multicenter cohort study
Source: Front Endocrinol (Lausanne). 2026 Jul 23;17:1866115. doi: 10.3389/fendo.2026.1866115 (PMC13441787; doi:10.3389/fendo.2026.1866115)
Supplement: Supplementary file 1 [file Supplementaryfile1.docx]

**Supplementary Materials**

**Table of Contents**

**Supplementary Methods**

**Supplementary Table S1.** ICD-10 codes and associated procedure codes used to identify CV diseases and ESKD

**Supplementary Table S2.** Matching matrix between different clustering model

**Supplementary Table S3.** Baseline characteristics of medication-excluded K-means clusters.

**Supplementary Table S4.** Agreement between original K-means clusters and medication-excluded feature-selected K-means clusters.

**Supplementary Table S5.** Age-standardized all-cause mortality rates according to diabetic CKD subtype.

**Supplementary Table S6.** One-, three-, and five-year cumulative incidence and absolute risk differences by diabetic CKD subtype, using the comorbidity-dominant subtype as the reference group.

**Supplementary Table S7.** Proportional hazards assumption assessment according to diabetic CKD subtype, using the comorbidity-dominant subtype as the reference group.

**Supplementary Table S8.** Time-stratified sensitivity analysis of clinical outcomes before and after 7 years of follow-up, using the comorbidity-dominant subtype as the reference group.

**Supplementary Table S9.** Incremental predictive performance after adding diabetic CKD subtype to Model 3.

**Supplementary Figure S1.** A flowchart of the study participant selection

**Supplementary Figure S2**. Overall workflow of the study analysis

**Supplementary Figure S3**. Davies-Bouldin index according to the number of clusters.

**Supplementary Figure S4.** PCA plot of K-means clustering (k=3)

**Supplementary Figure S5**. Agreement of cluster assignments between K-means clustering and other clustering methods

**Supplementary Figure S6.** Standardized mean differences of baseline characteristics across the three diabetic CKD subtypes

**Supplementary Methods**

***Dimensionality reduction and visualization***

Cluster separation was visualized using principal component analysis (PCA) and t-distributed stochastic neighbor embedding (t-SNE). Before analysis, all baseline variables were standardized using Z score transformation to ensure comparability across different measurement scales. PCA was applied to project the high-dimensional data onto two principal components that captured the largest sources of variance in the dataset. t-SNE was used to generate two-dimensional embeddings to visualize potential non-linear separation between clusters. These plots were used as descriptive visualization tools to examine the overall distribution of the identified clusters.^1^

***Internal validation using a subsampling consensus heatmap***

The stability and reproducibility of the K-means clustering results were evaluated using a subsampling consensus heatmap. In each of 1,000 bootstrap iterations, 80% of the dataset was randomly sampled without replacement, K-means clustering was reapplied to the subsample, and the co-clustering frequency for each pair of participants was recorded. Consensus values ranged from 0, indicating that two participants were never assigned to the same cluster, to 1, indicating that they were always assigned to the same cluster. The consensus heatmap was constructed to visualize the proportion of times each participant pair was grouped together across bootstrap samples, providing an internal measure of clustering robustness.

***Intermodel validation with hierarchical clustering***

Robustness of the K-means clustering solution was further assessed by comparing the primary K-means cluster assignments with those obtained using hierarchical clustering. Hierarchical clustering was conducted using agglomerative clustering with Ward’s linkage and Euclidean distance. Agreement between clustering methods was assessed using cross-tabulation and the adjusted Rand index (ARI). In addition, a Sankey plot was generated to visualize participant-level concordance across K-means and hierarchical clustering assignments.^1^

***Medication-excluded clustering sensitivity analysis***

To evaluate whether medication variables disproportionately influenced cluster formation, we performed a sensitivity analysis in which medication variables were excluded from the K-means input features. The same preprocessing strategy used in the primary clustering analysis was applied to the remaining demographic, comorbidity, and laboratory variables. The medication-excluded clustering solution was summarized using baseline characteristics and compared with the primary K-means clustering solution using cross-tabulation and ARI.

***Outcome analysis with competing risks***

The prognostic significance of each cluster was examined using outcome-specific survival models. For CV disease and progression to ESKD, Fine–Gray subdistribution hazard models were used, with death treated as a competing event.^2,3^ For all-cause mortality, Cox proportional hazards models were used. Three sequential models were fitted: Model 1 was unadjusted; Model 2 was adjusted for age, sex, BMI, hypertension, dyslipidemia, history of CV disease, and history of cancer; and Model 3 was additionally adjusted for eGFR, hemoglobin, HbA1c, LDL cholesterol, albumin, UPCR/proteinuria, and enrollment period. Hazard ratios or subdistribution hazard ratios with 95% CIs were estimated according to the reference group specified in each table.

***Additional outcome analyses and proportional hazards assessment***

For CV disease and progression to ESKD, cumulative incidence was estimated with death treated as a competing event. For all-cause mortality, cumulative mortality was estimated over follow-up. One-, three-, and five-year cumulative incidence estimates and absolute risk differences were calculated by diabetic CKD subtype, using the comorbidity-dominant subtype as the reference group. Risk differences were expressed as percentage points.

The proportional hazards assumption was assessed using Schoenfeld residual-based tests. For all-cause mortality, the test was applied to Cox proportional hazards models. For CV disease and progression to ESKD, which were evaluated using Fine–Gray subdistribution hazard models in the primary analysis, proportionality was assessed using cause-specific Cox models as diagnostic sensitivity models.^3^ Because possible non-proportionality was observed for some comparisons, a time-stratified sensitivity analysis was performed before and after 7 years of follow-up. For CV disease and progression to ESKD, cause-specific Cox Model 3 was used in the time-stratified sensitivity analysis; for all-cause mortality, Cox Model 3 was used.

***Age-standardized mortality analysis***

To provide context for the high all-cause mortality burden, age-standardized all-cause mortality rates were calculated by direct standardization.^4^ The person-year distribution of the entire analytic cohort was used as the standard population. Crude and age-standardized mortality rates were expressed per 1,000 person-years with 95% confidence intervals.

***Incremental predictive performance analysis***

The incremental predictive value of diabetic CKD subtype was assessed by comparing Model 3 with an expanded model that additionally included diabetic CKD subtype. Five-year prediction performance was evaluated using time-dependent AUC, continuous net reclassification improvement (NRI), and integrated discrimination improvement (IDI). For CV disease and progression to ESKD, NRI and IDI were calculated using Cox-based 5-year prediction models for consistency with the time-dependent ROC analyses. Positive NRI or IDI values were interpreted as improved risk reclassification or discrimination after adding diabetic CKD subtype to Model 3.

**References**

Dashtban A, Mizani MA, Pasea L, et al. Identifying subtypes of chronic kidney disease with machine learning: development, internal validation and prognostic validation using linked electronic health records in 350,067 individuals. *EBioMedicine* 2023; **89**: 104489.

Chen DC, Shlipak MG, Scherzer R, et al. Association of Intraindividual Difference in Estimated Glomerular Filtration Rate by Creatinine vs Cystatin C and End-stage Kidney Disease and Mortality. *JAMA Netw Open* 2022; **5**(2): e2148940.

Hassan W, Shrestha P, Sumida K, et al. Association of Uric Acid-Lowering Therapy With Incident Chronic Kidney Disease. *JAMA Netw Open* 2022; **5**(6): e2215878.

Mark PB, Stafford LK, Grams ME, et al. Global, regional, and national burden of chronic kidney disease in adults, 1990-2023, and its attributable risk factors: a systematic analysis for the Global Burden of Disease Study 2023. *The Lancet* 2025; **406**(10518): 2461–82.

**Supplementary Table S1.** ICD-10 codes and associated procedure codes used to identify CV diseases and ESKD

| **Conditions** | **ICD-10 codes** | **Procedure codes** |
| --- | --- | --- |
| Ischemic heart disease, myocardial infarction | I21-I22 | M6565, M6566, M6561, M6562, M6571, M6553, M6551, M6554, M6552, HA681, M6567, HA670, M6563, M6564 |
| Stroke | I60-64 | M6630, M6635, M6636, M6637, M6593, M6594, M6599, M6601, M6602 |
| ESKD | N18, Z49 | O7033, O7080, O7061, O7062, O7034, O7035, O7032, O7031, O7018, O2083, O7018, E6593, O7020, O9991 |

Abbreviation: ICD-10, 10th International Classification of Diseases; CV, cardiovascular; ESKD, end-stage kidney disease

**Supplementary Table S2.** Matching matrix between different clustering model

| **Concordance (%)** | | **K-means clustering** | | |
| --- | --- | --- | --- | --- |
|  |  | **Cluster 1** | **Cluster 2** | **Cluster 3** |
| **Hierarchical clustering** | Cluster A | 9.0 | 72.6 | 9.6 |
|  | Cluster B | 82.1 | 20.2 | 21.3 |
|  | Cluster C | 8.9 | 7.2 | 69.1 |

**Supplementary Table S3.** Baseline characteristics of medication-excluded K-means clusters.

| **Variables** | **Cluster 1 (n = 4,661)** | **Cluster 2 (n = 2,380)** | **Cluster 3 (n = 4,884)** | **P value*** |
| --- | --- | --- | --- | --- |
| **Demographics, n (%)** |  |  |  |  |
| Age |  |  |  | <0.001 |
| 19-29, n (%) | 13 (0.3) | 16 (0.7) | 49 (1.0) |  |
| 30-39, n (%) | 82 (1.8) | 74 (3.1) | 85 (1.7) |  |
| 40-49, n (%) | 239 (5.1) | 219 (9.2) | 303 (6.2) |  |
| 50-59, n (%) | 688 (14.8) | 426 (17.9) | 820 (16.8) |  |
| 60-69, n (%) | 1,515 (32.5) | 598 (25.1) | 1,634 (33.5) |  |
| ≥70, n (%) | 2,124 (45.6) | 1,047 (44.0) | 1,993 (40.8) |  |
| Sex |  |  |  | <0.001 |
| Male, n (%) | 2,476 (53.1) | 1,344 (56.5) | 3,007 (61.6) |  |
| Female, n (%) | 2,185 (46.9) | 1,036 (43.5) | 1,877 (38.4) |  |
| BMI (kg/m²) |  |  |  | <0.001 |
| <18.5, n (%) | 72 (1.5) | 136 (5.7) | 110 (2.3) |  |
| ≥18.5 and <23, n (%) | 1,361 (29.2) | 913 (38.4) | 1,491 (30.5) |  |
| ≥23 and <25, n (%) | 1,806 (38.7) | 728 (30.6) | 1,755 (35.9) |  |
| ≥25, n (%) | 1,422 (30.5) | 603 (25.3) | 1,528 (31.3) |  |
| **Comorbidity, n (%)** |  |  |  |  |
| Hypertension | 3,824 (82.0) | 1,397 (58.7) | 2,118 (43.4) | <0.001 |
| Dyslipidemia | 4,661 (100.0) | 987 (41.5) | 11 (0.2) | <0.001 |
| CVD | 1,178 (25.3) | 337 (14.2) | 763 (15.6) | <0.001 |
| Cancer | 209 (4.5) | 124 (5.2) | 496 (10.2) | <0.001 |
| **Laboratory data, n (%)** |  |  |  |  |
| eGFR (mL/min/1.73 m²) |  |  |  | <0.001 |
| ≥60, n (%) | 1,016 (21.8) | 345 (14.5) | 622 (12.7) |  |
| 45-60, n (%) | 2,156 (46.3) | 631 (26.5) | 2,453 (50.2) |  |
| <45, n (%) | 1,489 (31.9) | 1,404 (59.0) | 1,809 (37.0) |  |
| UPCR ≥300 mg/g | 2,464 (52.9) | 2,145 (90.1) | 3,216 (65.8) | <0.001 |
| **Laboratory data, mean (SD)** |  |  |  |  |
| HbA1c (%) | 7.4 ± 1.5 | 7.6 ± 2.0 | 7.4 ± 1.5 | 0.001 |
| Hb (g/dL) | 12.4 ± 2.0 | 9.9 ± 1.8 | 12.4 ± 1.9 | <0.001 |
| Leukocyte (10³/uL) | 7.5 ± 2.6 | 9.2 ± 7.0 | 7.4 ± 2.7 | <0.001 |
| Platelet (10³/uL) | 245.0 ± 81.5 | 233.1 ± 113.0 | 239.4 ± 85.0 | <0.001 |
| Albumin (g/dL) | 4.1 ± 0.4 | 3.1 ± 0.4 | 4.1 ± 0.3 | <0.001 |
| Sodium (mEq/L) | 139.6 ± 3.5 | 137.6 ± 5.4 | 139.6 ± 3.2 | <0.001 |
| Potassium (mEq/L) | 4.5 ± 0.6 | 4.5 ± 0.8 | 4.6 ± 0.6 | <0.001 |
| Chloride (mEq/L) | 104.1 ± 4.2 | 105.1 ± 6.4 | 104.5 ± 4.1 | <0.001 |
| Calcium (mg/dL) | 9.1 ± 0.5 | 8.2 ± 0.6 | 9.1 ± 0.5 | <0.001 |
| Phosphorus (mg/dL) | 3.6 ± 0.8 | 3.9 ± 1.3 | 3.6 ± 0.7 | <0.001 |
| Uric acid (mg/dL) | 6.3 ± 2.0 | 7.0 ± 2.5 | 6.5 ± 2.0 | <0.001 |
| Glucose (mg/dL) | 157.0 ± 83.8 | 171.9 ± 102.4 | 151.0 ± 76.2 | <0.001 |
| AST (U/L) | 32.2 ± 26.5 | 98.5 ± 51.3 | 25.1 ± 30.5 | <0.001 |
| ALT (U/L) | 26.8 ± 12.5 | 49.0 ± 21.9 | 23.3 ± 30.4 | <0.001 |
| LDL (mg/dL) | 88.1 ± 34.3 | 98.9 ± 49.6 | 99.2 ± 34.0 | <0.001 |
| **Medication, n (%)** |  |  |  |  |
| MFM | 1,970 (42.3) | 258 (10.8) | 693 (14.2) | <0.001 |
| SU | 1,461 (31.3) | 270 (11.3) | 727 (14.9) | <0.001 |
| DPP4i | 1,035 (22.2) | 250 (10.5) | 281 (5.8) | <0.001 |
| Insulin | 381 (8.2) | 309 (13.0) | 163 (3.3) | <0.001 |
| ARB or ACEi | 2,514 (53.9) | 605 (25.4) | 899 (18.4) | <0.001 |
| Diuretics | 1,367 (29.3) | 612 (25.7) | 544 (11.1) | <0.001 |
| Beta blocker | 1,009 (21.6) | 371 (15.6) | 265 (5.4) | <0.001 |
| Statin | 3,132 (67.2) | 555 (23.3) | 3 (0.1) | <0.001 |
| Anti-platelet | 2,480 (53.2) | 632 (26.6) | 891 (18.2) | <0.001 |

Abbreviation: ACEi, angiotensin converting enzyme inhibitor; ALT, alanine aminotransferase; ARB, angiotensin II receptor blocker; AST, aspartate aminotransferase; BMI, body mass index; Ca, calcium; Cl, chloride; CVD, cardiovascular disease; DPP4i, dipeptidyl peptidase 4 inhibitor; eGFR, estimated glomerular filtration rate; Hb, hemoglobin; HbA1c, hemoglobin A1c; LDL, low density lipoprotein cholesterol; MFM, metformin; SU, sulfonylurea; UPCR, urine protein to creatinine ratio.

* A P-value <0.001 indicates statistically significant difference among the clusters.

**Supplementary Table S4.** Agreement between original K-means clusters and medication-excluded feature-selected K-means clusters.

| **Original K-means subtype** | **New cluster 1, n (%)** | **New cluster 2, n (%)** | **New cluster 3, n (%)** |
| --- | --- | --- | --- |
| Comorbidity-dominant | 3293 (73.3%) | 432 (9.6%) | 769 (17.1%) |
| Advanced CKD | 186 (8.3%) | 1712 (76.4%) | 342 (15.3%) |
| Obesity-dominant | 1182 (22.8%) | 236 (4.6%) | 3773 (72.7%) |

Abbreviation: CKD, chronic kidney disease.

**Supplementary Table S5.** Age-standardized all-cause mortality rates according to diabetic CKD subtype.

| **Subtype** | **N** | **Deaths** | **Crude mortality rate per 1,000 person-years** | **Age-standardized mortality rate,**  **per 1,000 person-years (95% CI)** |
| --- | --- | --- | --- | --- |
| Comorbidity-dominant | 4494 | 2045 | 84.96 | 84.72 (80.86–88.58) |
| Advanced CKD | 2240 | 1254 | 100.04 | 105.79 (99.68–111.91) |
| Obesity-dominant | 5191 | 3403 | 81.87 | 82.17 (79.41–84.94) |

Abbreviations: CI, confidence interval; CKD, chronic kidney disease.

**Supplementary Table S6.** One-, three-, and five-year cumulative incidence and absolute risk differences by diabetic CKD subtype, using the comorbidity-dominant subtype as the reference group.

| **Outcome**^†^ | **Time point, years** | **Comorbidity-dominant cumulative incidence, %** | **Advanced CKD cumulative incidence, %** | **Obesity-dominant cumulative incidence, %** | **RD: Advanced CKD vs Comorbidity-dominant, percentage points**^‡^ | **RD: Obesity-dominant vs Comorbidity-dominant, percentage points**^‡^ |
| --- | --- | --- | --- | --- | --- | --- |
| CV disease | 1 | 14.09 | 15.35 | 18.17 | 1.26 | 4.08 |
|  | 3 | 19.62 | 22.62 | 24.02 | 3 | 4.4 |
|  | 5 | 24.87 | 28.11 | 28.98 | 3.24 | 4.11 |
| Progression to ESKD | 1 | 2.34 | 12.92 | 1.47 | 10.58 | -0.88 |
|  | 3 | 8.4 | 30.84 | 6.69 | 22.43 | -1.71 |
|  | 5 | 15.6 | 40.97 | 11.86 | 25.37 | -3.74 |
| All-cause mortality | 1 | 4.13 | 9.83 | 2.33 | 5.7 | -1.8 |
|  | 3 | 14.86 | 20.15 | 9.35 | 5.29 | -5.51 |
|  | 5 | 26.97 | 30.66 | 15.56 | 3.69 | -11.4 |

Abbreviations: CKD, chronic kidney disease; CV, cardiovascular; ESKD, end-stage kidney disease; RD, risk difference.

^†^For CV disease and progression to ESKD, death was treated as a competing event.

^‡^Risk differences are expressed as percentage points.

**Supplementary Table S7.** Proportional hazards assumption assessment according to diabetic CKD subtype, using the comorbidity-dominant subtype as the reference group.

| **Outcome*** | **Subtype** | **Test statistic** | **P value** |
| --- | --- | --- | --- |
| CV disease | Advanced CKD | 3.42 | 0.064 |
|  | Obesity-dominant | 6.56 | 0.010 |
| Progression to ESKD | Advanced CKD | 7.18 | 0.007 |
|  | Obesity-dominant | 50.68 | <0.001 |
| All-cause mortality | Advanced CKD | 4.59 | 0.032 |
|  | Obesity-dominant | 5.74 | 0.017 |

Abbreviations: CKD, chronic kidney disease; CV, cardiovascular; ESKD, end-stage kidney disease; PH, proportional hazards.

*PH assumption was assessed using Schoenfeld residual-based tests. For CV disease and progression to ESKD, PH assumption was assessed using cause-specific Cox models as a diagnostic sensitivity assessment. P values <0.05 suggest possible evidence of non-proportionality.

**Supplementary Table S8.** Time-stratified sensitivity analysis of clinical outcomes before and after 7 years of follow-up, using the comorbidity-dominant subtype as the reference group.

| **Outcome** | **Follow-up period** | **Subtype** | **Model 3 HR (95% CI)*** | **P value** |
| --- | --- | --- | --- | --- |
| CV disease | 0 to ≤7 years | Advanced CKD | 1.13 (0.99–1.30) | 0.069 |
|  |  | Obesity-dominant | 1.38 (1.25–1.52) | <0.001 |
|  | >7 years | Advanced CKD | 0.93 (0.63–1.38) | 0.719 |
|  |  | Obesity-dominant | 0.74 (0.55–0.98) | 0.038 |
| Progression to ESKD | 0 to ≤7 years | Advanced CKD | 1.35 (1.17–1.55) | <0.001 |
|  |  | Obesity-dominant | 0.79 (0.70–0.90) | <0.001 |
|  | >7 years | Advanced CKD | 1.04 (0.76–1.43) | 0.804 |
|  |  | Obesity-dominant | 1.13 (0.92–1.38) | 0.239 |
| All-cause mortality | 0 to ≤7 years | Advanced CKD | 0.79 (0.70–0.90) | <0.001 |
|  |  | Obesity-dominant | 0.59 (0.54–0.66) | <0.001 |
|  | >7 years | Advanced CKD | 0.72 (0.61–0.84) | <0.001 |
|  |  | Obesity-dominant | 0.75 (0.67–0.84) | <0.001 |

Abbreviations: CI, confidence interval; CKD, chronic kidney disease; CV, cardiovascular; ESKD, end-stage kidney disease; HR, hazard ratio.

*Model 3 was adjusted for age, sex, BMI, hypertension, dyslipidemia, history of CV disease, history of cancer, eGFR, hemoglobin, HbA1c, LDL cholesterol, albumin, UPCR/proteinuria, and enrollment period.

**Supplementary Table S9.** Incremental predictive performance after adding diabetic CKD subtype to Model 3.

| **Outcome** | **Continuous NRI (95% CI)** | **NRI P value** | **IDI (95% CI)** | **IDI P value** |
| --- | --- | --- | --- | --- |
| CV disease | 0.021 (-0.030–0.072) | 0.342 | 0.002 (0.001–0.003) | 0.001 |
| Progression to ESKD | 0.111 (0.057–0.164) | 0.001 | 0.006 (0.004–0.007) | 0.001 |
| All-cause mortality | 0.293 (0.245–0.339) | 0.001 | 0.007 (0.006–0.009) | 0.001 |

Abbreviations: CKD, chronic kidney disease; CV, cardiovascular; ESKD, end-stage kidney disease; IDI, integrated discrimination improvement; NRI, net reclassification improvement.

*NRI and IDI were calculated for 5-year risk prediction by comparing Model 3 with Model 3 plus diabetic CKD subtype.

**Supplementary Figure S1.** A flowchart of the study participant selection

**Supplementary Figure S2**. Overall workflow of the study analysis

**Supplementary Figure S3**. Davies-Bouldin index according to the number of clusters.

**Supplementary Figure S4.** PCA plot of K-means clustering (k=3)

Abbreviation: PCA, principal component analysis

**Supplementary Figure S5**. Concordance of cluster assignments between K-means clustering and hierarchical clustering

**Supplementary Figure S6.** Standardized mean differences of baseline characteristics across the three diabetic CKD subtypes
